# Draft genome sequence of Elizabeth River PAH-degrading fungal isolates *Trichoderma deliquescens* and *Aureobasidium* sp.

**DOI:** 10.1128/mra.01089-25

**Published:** 2026-04-02

**Authors:** Erica Babusci, Claudia K. Gunsch

**Affiliations:** 1 Department of Civil and Environmental Engineering, Duke University 3065, Durham, North Carolina, USA; University of Maryland School of Medicine, Baltimore, Maryland, USA

**Keywords:** Genomics, *Trichoderma deliquescens*, Nanopore long-read sequencing, *Aureobasidium* sp., PAH degradation, Funannotate

## Abstract

Here, we report the draft genome sequences of *Trichoderma deliquescens* and *Aureobasidium* sp., fungi isolated from sediments of the Elizabeth River. Genomes assembled from Nanopore sequencing revealed genes encoding enzymes implicated in polycyclic aromatic hydrocarbon (PAH) degradation. These resources provide genomic insights into fungal PAH degradation and inform bioremediation strategies.

## ANNOUNCEMENT

*Trichoderma deliquescens* is a filamentous fungus, whereas *Aureobasidium* sp. is a yeast-like fungus (1, 2). Both of these species were previously isolated from the Elizabeth River sediment using sterile plastic bags in regions of heavy polycyclic aromatic hydrocarbon (PAH) contamination and validated for their PAH degradation ability (3–5). Here, we report the draft genome sequences of these strains to investigate whether morphological differences correspond to genomic features underlying PAH degradation capacity. Our objective is to provide insights into fungal biology and bioremediation potential through functional genome annotation.

Both fungi were cultured on malt extract agar (Difco, Detroit, MI) plates at 30°C for 5 days. Genomic DNA was extracted from 250 mg of homogenized agar using the DNeasy Soil Power Pro kit (Qiagen, Germantown, MD; cat. no. 47016). Species identity was confirmed by amplifying and sequencing the ITS/LSU region using the primers and protocols described in Sting et al. as ITS1-modified (5′–TCCGTWGGTGAACCWGCGG–3′) and NL4-modified (5′–GGTCCGTGTTTCAAGACGGG–3′) (6), which showed 97.63% similarity to *T. deliquescens* and 99.52% similarity to *Aureobasidium* sp. based on NCBI blastn analysis.

Libraries were prepared with the Oxford Nanopore Technologies (ONT) Rapid Barcoding Kit 24 V14 (SQK-RBK114.24) and sequenced on the PromethION 2 Solo with a Flow Cell R10 (M version; FLO-PROM114M) using MinKNOW v24.11.10. Raw reads were basecalled and demultiplexed using a dorado pipeline (Gitlab Project ID: 42374). Seqkit (v2.10.0) reported that *T. deliquescens* run generated 30,877 reads with an N50 of 1,223.7 bp, while the *Aureobasidium* sp. run produced 30,347 reads with an N50 of 1,249 bp. Reads were assembled with Flye v2.9.5 and polished with Medaka v2.0.1(7). The *T. deliquescens* assembly spanned 35,967,837 bp across 59 contigs with an N50 of 2,804,537 bp, a GC content of 52.14%, and a BUSCO completeness of 98.9%. The *Aureobasidium* sp. assembly spanned 27,536,729 bp across 23 contigs with an N50 of 2,191,637 bp, a GC content of 50.05%, and a BUSCO completeness of 97.8% (8, 9). Default parameters were used, unless stated otherwise.

Annotation was performed with Funannotate v1.8.16, which predicted 9,488 genes in *T. deliquescens* and 9,629 genes in *Aureobasidium* sp (10). InterPro Scan v5.73-104.0 identified protein domains, which were filtered for common PAH degradation enzymes (11). Comparative analysis revealed 124 PAH-related genes in *T. deliquescens*, while *Aureobasidium* sp. encoded for 126. While there were clear differences in the genome size and complexity, they still possess similar capacities, indicating both are viable options for bioremediation.

By comparing the genome size, gene content, and the composition of degradation-related enzymes, this work provides a framework for linking fungal morphology to functional potential for remediation applications.

## Data Availability

The genome sequences and annotations reported here were deposited in the National Center for Biotechnology Information (NCBI) GenBank sequence database under accession numbers JBRFVR010000000.1 and JBRFVQ000000000.1 for *T. deliquescens* and *Aureobasidium* sp., respectively. Raw, unfiltered fastQ sequences were submitted to the NCBI Sequence Resource Archive under BioProject accession numbers SRR36018447 and SRR36018446.
